# Workplace gender composition and long‐term sickness absence due to mental disorders: A retrospective cohort study

**DOI:** 10.1002/pcn5.70158

**Published:** 2025-07-14

**Authors:** Shohei Okura, Shinichi Iwasaki, Yasuhiko Deguchi, Yuki Kageyama, Kunio Maekubo, Ayaka Matsunaga, Koki Inoue

**Affiliations:** ^1^ Department of Neuropsychiatry Graduate School of Medicine, Osaka Metropolitan University Osaka Japan

**Keywords:** adjustment disorder, gender composition, long‐term sickness absence, major depressive disorder, mental disorders

## Abstract

**Aim:**

Long‐term sickness absence (LTSA) due to mental disorders is a public health concern. Workplace gender composition may influence employees' mental health outcomes; however, its impact on LTSA remains unclear. This study examined the association between workplace gender composition and the risk of LTSA due to common mental disorders, including major depressive disorder, adjustment disorder, and depressive state.

**Methods:**

This was a retrospective cohort study of Japanese public employees who applied for LTSA for mental disorders between 2011 and 2022. Workplace gender composition or the proportion of female employees was used as the exposure variable. Multinomial logistic regression was used to estimate the relative risk ratio (RRR) of LTSA due to major depressive disorder, depressive state, and adjustment disorder.

**Results:**

Of the 943 employees, 55.7% applied for LTSA due to major depressive disorder; 23.2%, adjustment disorder; and 21.1%, depressive state. Multinomial logistic regression showed that major depressive disorder was associated with age (RRR = 1.03, 95% confidence interval [CI]: 1.01–1.05), gender composition (RRR = 0.43, 95% CI: 0.19–0.95), manager (RRR = 0.61, 95% CI: 0.37–0.99), and clerical worker (RRR = 1.72, 95% CI: 1.05–2.83). Male‐dominated workplaces were associated with a higher relative risk of LTSA due to major depressive disorder. In contrast, female‐dominated workplaces were associated with a higher relative risk of LTSA due to adjustment disorders.

**Conclusion:**

Workplace gender composition influences LTSA due to mental disorders. Therefore, mental health strategies and interventions should be tailored to the gender composition of a workplace.

## INTRODUCTION

Mental disorders not only diminish one's quality of life, but also impose substantial economic burdens, including increased healthcare costs and reduced productivity.^1^, ^2^ Moreover, common mental disorders, which are frequent in occupational settings, influence sickness absence three times more than physical diseases.^3^ In recent years, the increase in long‐term sickness absence (LTSA) has become a significant social concern, with mental disorders as its primary contributing factor.^4^ Additionally, LTSA is associated with reduced labor force and financial losses for organizations, making its prevention and management global public health and occupational concerns.^5^

Consequently, there is growing interest in the impact of workplace environment on employee health, with workplace gender composition as a key factor. Several studies have examined this association and suggested that workplace gender composition contributes to health. Belonging to a minority gender negatively impacts health in the workplace.^6^, ^7^ For example, men working in female‐dominated workplaces exhibit poor self‐rated health.^8^ By contrast, psychological distress is higher in workplaces that are gender neutral than in those with a clear gender majority.^9^ Evidently, these findings are inconsistent, and further investigation is required to clarify the impact of workplace gender composition on health.

The relationship between workplace gender composition and sickness absence has been explored in multiple studies. Some studies have indicated that sickness absence is higher in workplaces that have authority of a majority gender and employees of a minority gender.^10^, ^11^ Conversely, some have shown that women have the highest number of sickness absences in female‐dominated workplaces.^12^ Similarly, male employees working in male‐dominated, high‐stress environments have a higher likelihood of taking sick leave than those working in other workplace settings.^13^ Therefore, these inconsistent findings also warrant further research to clarify the impact of workplace gender composition on sickness absence. Moreover, the underlying mechanisms of the impact of gender composition on sickness absence are unclear.

Workplace policies addressing mental health challenges are uniformly implemented irrespective of gender composition. Furthermore, previous studies have primarily focused on sickness absences, and no research has specifically investigated the relationship between workplace gender composition and mental disorders that cause sickness absences. A clearer understanding of this relationship is crucial from a public health perspective as it may contribute to the development of targeted interventions aimed at promoting mental health, preventing mental disorders, and enhancing workplace productivity. Among common mental disorders, major depressive disorder, depressive state, and adjustment disorders differ in terms of etiological factors, treatment strategies, and environmental adjustments. Therefore, examining the relationship between specific disorders and workplace factors is highly valuable for developing effective support measures.

Accordingly, this study aimed to clarify the differences in the impact of workplace gender composition on LTSA due to mental disorders. Our previous study has reported that employees of any gender working in female‐dominated workplaces exhibit higher occupational stress than those in other workplaces.^14^ Therefore, we hypothesized that employees working in female‐dominated workplaces have a higher risk of LTSA due to major depressive disorder, a common mental disorder, compared to those in male‐dominated workplaces.

## METHODS

### Study design

This research employed a retrospective cohort design.

### Participants' demographics

This study included public employees who took an LTSA due to mental disorders for 3 months or longer between 2011 and 2022 while working at city hall or ward offices in City A in the Kansai region of Japan. The following three most common mental conditions leading to LTSA were included: major depressive disorder, adjustment disorder, and depressive state. These are commonly observed in occupational settings and are generally classified as mental disorders.^15^ After receiving secondary data containing encrypted IDs of 1729 employees who took LTSA (0.7% of the total, 1729/236,172), the data were anonymized by administrative staff in City A. Of these, data of 943 employees (54.5%) were included in the analysis, excluding those of 494 due to physical illnesses and 292 due to mental disorders other than those included in this study.

### Sociodemographic variables

Gender and age were the demographic variables, whereas job rank (non‐manager and manager) and job category (clerical, technical, and professional workers) were occupational variables. Clerical workers were defined as individuals engaged in clerical tasks related to the construction, design, and management (among various roles) of buildings in the municipality; technical workers were those involved in technical tasks requiring physical effort in the municipality; and professional workers included nurses, care workers, public health nurses, and childcare workers.

### Diagnosis of sickness absence

The diagnosis in the medical certificate submitted to the workplace was used as the reason for LTSA. In cases of multiple diagnoses, the first was considered as the primary. However, these diagnoses were not necessarily based on the *International Classification of Diseases*, Tenth Revision (ICD‐10).^16^ Therefore, in this study, the medical certificates for each LTSA were reviewed by researchers or physicians with more than 10 years of experience and classified according to the relevant ICD‐10 codes. Thus, the most common mental disorders in the workplace—major depressive disorder (F320–F328), depressive state (F329), and adjustment disorder (F43)—were identified. Although major depressive disorder (F320–F328) and depressive state (F329) belong to the same diagnostic category, they were analyzed separately because depressive state is frequently used in medical certificates in Japan, reflecting a unique characteristic of Japanese clinical practice.

### Gender composition

Workplace gender composition was defined as an exposure variable based on the assumption that gender distribution in the target population remained relatively stable and did not fluctuate frequently over short periods. Workplace gender composition was quantified as the proportion of female employees in the target population, ranging from 0 to 1. For example, if female employees comprised 30% of the workplace, the value was 0.3.

### Statistical analyses

An independent *t*‐test or *χ* ^2^ test was used to compare gender, age, job title, job category, gender composition, and diagnosis. Furthermore, using adjustment disorder as the reference category, a multinomial logistic regression analysis was performed to identify the risk factors associated with LTSA due to major depressive disorder or depressive state. The results were presented as relative risk ratios (RRRs) with 95% confidence intervals (CIs), and *p*‐values were reported to determine statistical significance. Statistical significance was set at *p* < 0.05, and the analyses were performed using IBM SPSS Version 29.0 (IBM).

## RESULTS

Table 1 shows the participants’ characteristics (*n* = 943). In total, 66.0% (*n* = 622) were men and 34.0% (*n* = 321) were women. The participants' mean age (± standard deviation) was 43.6 ± 9.3 years. Among the participants, 86.0% (*n* = 811) were non‐managers and 14.0% (*n* = 132) were managers. Regarding job categories, 65.8% (*n* = 621) were clerical workers, 18.9% (*n* = 178) were technical workers, and 15.3% (*n* = 144) were professional workers. The mean gender composition (± standard deviation) was 0.36 ± 0.25. Regarding LTSA reasons, 55.7% (*n* = 525) reported major depressive disorder; 23.2% (*n* = 219), adjustment disorder; and 21.1% (*n* = 199), depressive state. All variables differed significantly between women and men.

**Table 1:** Participants’ characteristics and mental disorder diagnoses.

Variables	Range	Total	Women	Men	*p*
Gender		943	321 (34.0%)	622 (66.0%)	
Age (years)	19–64	43.6 ± 9.3	40.9 ± 9.8	44.9 ± 8.8	**
Job title					**
Non‐manager		811 (86.0)	295 (91.9)	516 (83.0)	
Manager		132 (14.0)	26 (8.1)	106 (17.0)	
Job category					**
Clerical worker		621 (65.8)	183 (57.0)	438 (70.4)	
Technical worker		178 (18.9)	34 (10.6)	143 (23.2)	
Professional worker		144 (15.3)	104 (32.4)	40 (6.4)	
Gender composition	0–1	0.36 ± 0.25	0.53 ± 0.26	0.28 ± 0.20	**
Diagnosis					*
Major depressive disorder		525 (55.7)	161 (50.2)	364 (58.5)	
Adjustment disorder		219 (23.2)	86 (25.2)	138 (22.2)	
Depressive state		199 (21.1)	79 (24.6)	120 (19.3)	

*Note*: An independent *t*‐test or chi‐square test was used to examine the differences between gender, age, job title, job category, gender composition, and diagnosis.

*p < 0.05

**p < 0.01.

Table 2 presents RRRs for LTSA due to major depressive disorder and depressive state, with adjustment disorder as the reference category. Workplace gender composition was significantly associated with LTSA due to major depressive disorder, with workplaces having a higher proportion of women being associated with a lower risk (RRR = 0.43, 95% CI: 0.19–0.95). Age was also significantly associated with LTSA due to major depressive disorder (RRR = 1.03, 95% CI: 1.01–1.05), indicating an increased risk with older age. Job title was another significant factor, with managers having a lower risk of LTSA due to major depressive disorder compared to non‐managers (RRR = 0.61, 95% CI: 0.37–0.99). Additionally, job category was associated with LTSA due to major depressive disorder, with clerical workers having a significantly higher risk than professional workers (RRR = 1.72, 95% CI: 1.05–2.83), while no significant difference was observed for technical workers. Gender was not significantly associated with LTSA for major depressive disorder. Furthermore, no significant associations were found for LTSA due to depressive state across any of the examined factors. These findings suggest that age, workplace gender composition, job title, and job category are significant factors in LTSA due to major depressive disorder, whereas none of these are associated with LTSA due to depressive state.

**Table 2:** The relative risk ratio of LTSA due to major depressive disorder, depressive state, and adjustment disorder.

	Major depressive disorder			Depressive state		
	RRR	(95% CI)	*p*	RRR	(95% CI)	*p*
Age	1.03	(1.01–1.05)	*	0.99	(0.96–1.01)	
Gender composition	0.43	(0.19–0.95)	*	0.54	(0.21–1.41)	
Gender						
Woman	1.11	(0.74–1.66)		1.35	(0.84–2.17)	
Man	Ref			Ref		
Job title						
Manager	0.61	(0.37–0.99)	*	1.16	(0.65–2.07)	
Non‐manager	Ref			Ref		
Job category						
Clerical worker	1.72	(1.05–2.83)	*	1.32	(0.74–2.39)	
Technical worker	1.29	(0.68–2.46)		1.04	(0.47–2.30)	
Professional worker	Ref			Ref		

*Note*: The reference category is adjustment disorder.

Abbreviations: CI, confidence interval; LTSA, long‐term sickness absence; Ref, reference category; RRR, relative risk ratio.

*p < 0.05.

## DISCUSSION

This study examined the association between workplace gender composition and the risk of LTSA due to common mental disorders. Contrary to our hypothesis, working in a male‐dominated workplace was associated with a higher relative risk of LTSA due to major depressive disorder than working in a female‐dominated workplace. In contrast, working in a female‐dominated workplace was associated with a higher relative risk of LTSA due to adjustment disorders than working in a male‐dominated workplace.

The relative risk of LTSA due to major depressive disorder was higher than that due to adjustment disorders in male‐dominated workplaces. According to the *Diagnostic and Statistical Manual of Mental Disorders*, Fifth Edition (DSM‐5),^17^ major depressive disorder is characterized by severe emotional and physical symptoms, with core features including depressed mood and loss of interest or pleasure. It significantly impairs functioning across key domains and may not be linked to specific stressors. Its etiology is unclear in many cases. In contrast, an adjustment disorder is diagnosed when an individual does not meet the criteria for major depressive disorder or other psychiatric disorders. It is a transient response to an identifiable stressor, with symptoms emerging within 3 months of the onset of the stressor and typically resolving within 6 months after its removal. Therefore, major depressive disorder is generally regarded as a more severe condition than adjustment disorder, with more profound symptoms and a greater impact on daily functioning. Owing to the complexity of treatment and the need for long‐term management, major depressive disorder is considered a serious psychiatric condition. Furthermore, a depressive state is considered a clinical condition rather than a distinct diagnosis. It is commonly used as a provisional diagnosis when the diagnostic criteria for major depressive or adjustment disorders are not fully met. In Japan, the term “depressive state” is frequently used in medical certificates and other documents submitted to workplaces.^18^ This may reflect physicians' concerns that stating a formal psychiatric diagnosis could lead to social disadvantages for the patient. However, the clinical characteristics of depressive state remain unclear. The category may encompass a wide range of mental conditions that do not belong to major depressive disorder or adjustment disorder, such as affective instability associated with developmental or personality disorders. To the best of our knowledge, no prior studies have directly compared depressive state and major depressive disorder. This diagnostic ambiguity appears to be a Japan‐specific issue rooted in practical clinical conventions. Therefore, distinguishing between depressive state, major depressive disorder, and adjustment disorder in this study represents a meaningful attempt to reflect the realities of clinical practice in Japan.

In this study, workers in male‐dominated workplaces were associated with a higher relative risk of LTSA due to major depressive disorder than those in female‐dominated workplaces. Previous research has reported that workers in male‐dominated workplaces have a significantly lower risk of cumulative sickness absence (≥ 90 days) compared to those in female‐dominated workplaces.^19^ While shift work was associated with mild depressive symptoms in male‐dominated occupations, it predicted an increase in LTSA in female‐dominated occupations.^20^ Therefore, the lower risk of LTSA in male‐dominated workplaces may be partly explained by workplace culture. Specifically, men tend to perceive sickness absence more negatively than women^21^ and have lower healthcare utilization rates, including fewer visits to primary care providers.^22^ This tendency may discourage taking leave or seeking support. Consequently, symptoms may worsen, and individuals may already meet the diagnostic criteria for major depressive disorder by the time they seek medical attention. This tendency appears to be more pronounced in male‐dominated workplaces where seeking support for mental health concerns or reporting psychological distress may be particularly challenging. Consequently, stress and psychological strain may accumulate, resulting in sickness absence at more severe stages of the disease. Previous studies have shown that employees in many male‐dominated occupations exhibit higher levels of depressive symptoms.^23^ This suggests that in male‐dominated workplaces, mental health issues are less likely to surface and seeking appropriate support is more challenging, ultimately leading to the accumulation of depressive symptoms. These factors explain why our study found an increased risk of LTSA due to major depressive disorder in male‐dominated workplaces.

In contrast, workers in female‐dominated workplaces were associated with a higher relative risk of LTSA due to adjustment disorders than those in male‐dominated workplaces. Female‐dominated workplaces may provide an environment in which employees feel more comfortable discussing their health concerns, leading them to opt for sickness absences early and preventing the progression of severe diseases. Women are more likely to access healthcare services than men.^21^, ^22^ In female‐dominated workplaces, both men and women have a higher risk of taking sickness absence for 14 days or more.^24^ Additionally, those with high rates of sickness absence tended to include a larger proportion of female and older employees.^25^ These findings may have been influenced by differences in working conditions. Specifically, occupations with a high proportion of female employees are more likely to involve shift work and part‐time positions with limited control over tasks and reduced workplace flexibility.^26^ Furthermore, female‐dominated organizations often experience unclear goals and job responsibilities, inadequate resources and staffing, insufficient managerial support, and an imbalance between job demands and control.^27^ These environmental factors may contribute to the accumulation of stress and increase the risk of sick leave. However, they may also facilitate early recognition of mental health issues, increasing the likelihood of seeking medical attention at milder stages of illness. Therefore, more cases may be diagnosed as adjustment disorders, potentially preventing progression to more severe conditions.

In this study, age, job title, and job category were significantly associated with LTSA due to mental disorders. Specifically, older employees had a higher risk of LTSA due to major depressive disorder. In general, major depressive disorder tends to have a higher incidence among older age groups than adjustment disorders.^28^ Older employees may experience reduced adaptability and flexibility in the workplace. Age‐related health issues or changes in social roles, such as retirement or social isolation, may negatively affect their mental health.^29^ These factors may explain the increased risk of LTSA due to major depressive disorder with age.

Furthermore, managers had a lower risk of LTSA due to major depressive disorder than non‐managers. Previous studies have shown that while managerial roles are associated with high job demands and work–life interference, they also provide greater influence, autonomy, job satisfaction, and overall life satisfaction, which are linked to lower absenteeism.^30^ These characteristics suggest that managers may have access to more effective stress‐reduction coping mechanisms, leading to a lower risk of developing major depressive disorder.

Additionally, clerical workers had a higher risk of LTSA due to major depressive disorder than professional workers. Prior research has indicated that while professional workers report higher levels of perceived stress and psychological demands than nonprofessional workers, their overall occupational stress tends to be lower.^31^ Professional occupation is also associated with better mental health status, higher job satisfaction, lower prevalence of mental disorders, greater job stability, and higher levels of autonomy. These findings align with the observations of this study, suggesting that professional workers possess protective factors that help mitigate workplace stress and may have a lower risk of developing major depressive disorder.

### Implications

The findings of this study indicate that workplace gender composition influences the risk of LTSA due to major depressive and adjustment disorders. Therefore, implementing support programs tailored to gender composition may contribute to the prevention of LTSA. For example, in male‐dominated workplaces, access to mental health support should be improved and barriers to seeking help should be reduced by providing health information and consultations with healthcare professionals. Additionally, early intervention through stress‐check programs to identify those with high stress and arrange follow‐up consultations is crucial. Furthermore, interventions using smartphone applications based on cognitive behavioral therapy and mindfulness may be effective in preventing depression and overall symptoms among employees who experience moderate to high levels of stress.^32^ These approaches enable employees to address their symptoms before they worsen, potentially reducing the risk of LTSA due to major depressive disorder. However, in female‐dominated workplaces, support should focus on improving working conditions and organizational culture, such as increasing flexibility in shift schedules and task assignments, enhancing managerial support, and ensuring adequate resources. These measures may help reduce workplace stress and lower the risk of developing adjustment disorders. Implementing appropriate interventions based on workplace gender composition may promote early diagnosis and management of mental health issues, ultimately contributing to a reduction in the risk of LTSA. Further empirical research is required to generalize these findings and translate them into practical interventions.

### Strengths and limitations

This study had several limitations. First, as the study population consisted exclusively of Japanese public employees, the external validity of the findings may be limited when applied to other countries or cultural contexts. Second, the gender distribution within the study sample was skewed, with men accounting for 66.0% of all participants. Third, primary care physicians diagnosed the issues, raising the possibility that alternative, less burdensome diagnostic labels may have been used. Previous research has suggested that the diagnoses recorded on sickness absence certificates may not fully reflect the complexity and comorbidities observed in structured psychiatric interviews.^33^ Fourth, several important confounding variables, such as marital/family status and educational attainment, were unavailable in the dataset and therefore not included in the analysis. As these factors are known to be associated with mental health outcomes and LTSA risk,^34^ their absence may have resulted in residual confounding, which should be considered when interpreting the findings. Fifth, although this study distinguished between major depressive disorder and adjustment disorder based on ICD‐10 codes, we acknowledge that these diagnoses often lie on a clinical continuum and that diagnostic boundaries may shift over time. This study was based on ICD‐10 codes recorded in medical certificates issued by attending physicians at the start of sickness absence; consequently, we were unable to track diagnostic changes longitudinally. In addition, the dataset did not include information on comorbid anxiety disorders, limiting our ability to account for the potential influence of psychiatric comorbidities.

Despite these limitations, this study has several strengths. To the best of our knowledge, this is the first study to examine the differences in LTSA diagnoses based on workplace gender composition. Additionally, this study employed a methodology in which workplace gender composition was calculated using registered employee data for each workplace. This approach provides a reliable representation of actual workplace conditions. Additionally, the study population was part of a large cohort and the study period spanned over 10 years, allowing for sufficient data collection. This enabled a more precise analysis of the relationship between workplace gender composition and LTSA due to mental health disorders.

## CONCLUSION

This study examined the impact of workplace gender composition on LTSA caused by mental disorders. The results revealed that the risk of an LTSA due to major depressive disorder was higher in male‐dominated workplaces, whereas that due to adjustment disorders was higher in female‐dominated workplaces, suggesting the roles of workplace culture and supporting environments. In male‐dominated workplaces, employees may face challenges in seeking mental health support, leading to delayed intervention and an increased likelihood of LTSA at a more advanced stage of the disease. Conversely, in female‐dominated workplaces, employees are more likely to recognize early signs of mental health issues and take LTSA at a milder stage. Therefore, strengthening workplace mental health support that is tailored to gender composition is essential.

## AUTHOR CONTRIBUTIONS

*Conceptualization*: Shohei Okura and Shinichi Iwasaki. *Methodology*: Shohei Okura and Shinichi Iwasaki. *Data collection*: Shinichi Iwasaki. *Data analysis*: Shohei Okura. *Writing—original draft*: Shohei Okura. *Writing—review and editing*: Shinichi Iwasaki, Yasuhiko Deguchi, Yuki Kageyama, Kunio Maekubo, and Ayaka Matsunaga. *Supervision*: Koki Inoue. All authors reviewed and approved the final version of the manuscript.

## CONFLICT OF INTEREST STATEMENT

The authors declare no conflicts of interest.

## ETHICS APPROVAL STATEMENT

The Human Subjects Review Committee of Osaka Metropolitan University approved the study protocol (authorization No. 3337).

## PATIENT CONSENT STATEMENT

As the study used secondary data that could be obtained anonymously, the review committee did not require written informed consent. To address potential concerns of the participants regarding the use of sensitive personal documents, information about this study was included on our department's website and the opportunity to opt out of the study was provided. In addition to anonymization, data were stored on secure servers and access was restricted to authorized personnel only, ensuring compliance with national data‐protection regulations. All methods were performed in accordance with relevant guidelines and regulations.

## CLINICAL TRIAL REGISTRATION

N/A.

## Data Availability

Data sharing not applicable to this article as no datasets were generated or analyzed during the current study.
